# *Bryophyllum pinnatum* in the treatment of restless legs syndrome: A case series documented with polysomnography

**DOI:** 10.1002/ccr3.2144

**Published:** 2019-04-14

**Authors:** Sigrid von Manitius, Dominique Flügel, Bettina Gievers Steinlein, Martin Schnelle, Ursula von Mandach, Ana Paula Simões‐Wüst

**Affiliations:** ^1^ Department of Neurology Kantonsspital St. Gallen St. Gallen Switzerland; ^2^ Weleda AG Arlesheim Switzerland; ^3^ Department of Obstetrics Zurich University Hospital Zurich Switzerland; ^4^ Research Department Clinic Arlesheim Arlesheim Switzerland

**Keywords:** *Bryophyllum pinnatum*, periodic limb movements during sleep, polysomnography, restless legs syndrome, sleep quality

## Abstract

Restless legs syndrome may seriously affect patients' sleep and quality of life, but established pharmacological therapy can often have severe side effects. Therefore, new therapeutic approaches such as well‐tolerated preparations from the medicinal plant *Bryophyllum pinnatum* should be considered as alternatives. Their sedative and spasmolytic properties might contribute to improve patients' condition.

## 1 INTRODUCTION

We set out to experimentally treat restless legs syndrome patients with preparations of *Bryophyllum pinnatum* that have been proposed to possess sedative and muscle relaxant properties. The disease courses of our five patients suggest that treatment with these preparations (mainly chewable tablets) can improve restless legs syndrome and sleep quality.

Restless legs syndrome (RLS) is a neurologic disorder characterized by a compelling urge to move one's legs, usually worsening in the evening and at night.^1^ The diagnosis is done clinically, based on five essential criteria (see Box ).1 Treatment of RLS is challenging. Patients often suffer from disturbed quality of life, whereby sleep problems play a marked role. These can be severe, include insomnia, and are often associated with additional features such as repetitive leg jerks, that is involuntary and stereotyped movements during sleep, the so‐called periodic limb movements during sleep (PLMS). Current pharmacological treatment strategies for RLS include dopaminergic agents (L‐Dopa or dopamine agonists has), α2δ calcium channel ligands, opioids, and benzodiazepines, all of which, however, frequently have marked side effects, especially in long‐term treatment (see eg Ref.^2^, ^3^). Whereas originally a positive response to L‐Dopa or dopamine agonists was considered an additional criterion for the diagnosis of RLS,^4^ and dopamine agonists were used as first‐line treatment, nowadays dopaminergic agents are known to cause augmentation and compulsive behavior.^3^ Sleepiness and weight gain are typical side effects with α2δ ligands, and dependency and daytime somnolence are always risks in treatment with opioids and benzodiazepines.

Box 1 **Diagnostic criteria for restless legs syndrome according to Allen et al^1^**

1

An urge to move the legs usually but not always accompanied by, or felt to be caused by, uncomfortable and unpleasant sensations in the legs.
The urge to move the legs and any accompanying unpleasant sensations begin or worsen during periods of rest or inactivity such as lying down or sitting.
The urge to move the legs and any accompanying unpleasant sensations are partially or totally relieved by movement, such as walking or stretching, at least as long as the activity continues.
The urge to move the legs and any accompanying unpleasant sensations during rest or inactivity only occur or are worse in the evening or night than during the day.
The occurrence of the above features is not solely accounted for as symptoms primary to another medical or a behavioral condition (eg myalgia, venous stasis, leg edema, arthritis, leg cramps, positional discomfort, habitual foot tapping).

Preparations of *Bryophyllum pinnatum* (BP) leaves have been proposed to possess both sedative and muscle relaxant properties.^5^, ^6^ Moreover, prospective observational trials performed with pregnant women^7^ and cancer patients^8^ revealed good effectiveness in the treatment of sleep disorders with BP 50% chewable tablets (each 350 mg tablet corresponds to 170 mg of leave press juice, dried down to 17 mg by mixing with lactose; 100 mg dried BP matter in 1 g). Therefore, we set out to experimentally treat RLS patients with this herbal preparation.

## 2 CASE PRESENTATION

**Figure 1:**
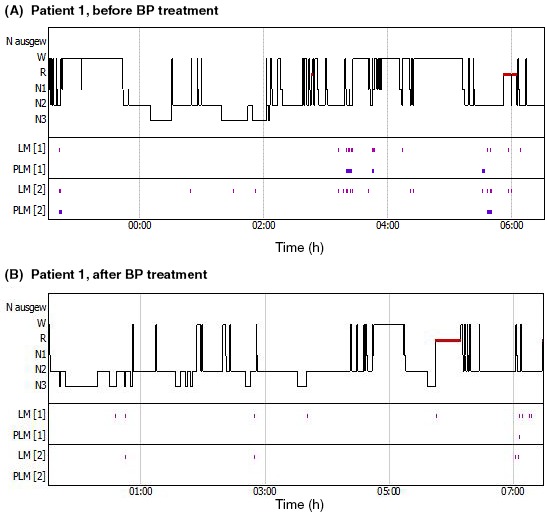
Polysomnography records of patient 1, who presented with low sleep efficiency, before and after treatment with *Bryophyllum pinnatum* 50% tablets. LM: limb movements (0.5‐10 s with an increase of electromyogram amplitude of ≥8 µV, compared to electromyogram during rest); PLM, period limb movements (at least four limb movements between 0.5 and 5 s, recurring at intervals of 5‐90 s); [1], right limb; [2], left limb

**Figure 2:**
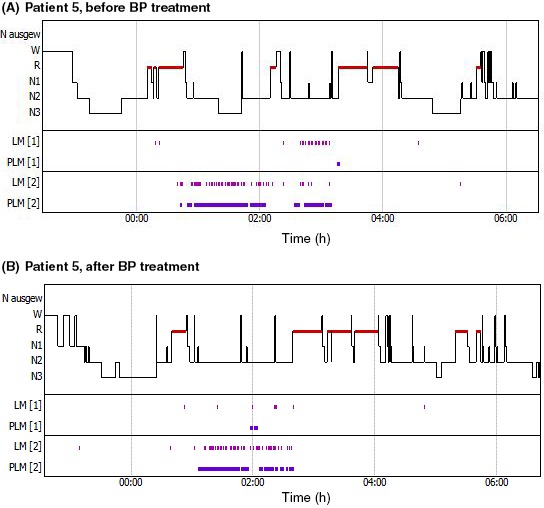
Polysomnography records of patient 5, who presented with frequent PLMS, before and after treatment with *Bryophyllum pinnatum* 33% mother tincture. LM: limb movements (0.5‐10 s with an increase of electromyogram amplitude of ≥8 µV, compared to electromyogram during rest); PLM, period limb movements (at least four limb movements between 0.5 and 5 s, recurring at intervals of 5‐90 s); [1], right limb; [2], left limb

In this consecutive case series, five patients with RLS, diagnosed according to the updated IRLSSG consensus criteria (see Box ),^1^ and poor sleep quality documented by polysomnography (PSG), were experimentally treated with BP preparations. Iron deficiency was ruled out in all patients by determining iron, ferritin, and transferrin levels before treatment begin. Brief case descriptions are presented below. The typical sequence of events was as follows: (a) patient presented at the hospital for consultation, the need for PSG for diagnostic purposes was perceived, a (baseline) PSG was performed some weeks later; (b) baseline PSG results were communicated to the patient in a second consultation and further treatment was discussed; (c) treatment with BP was initiated unless the introduction or dose adjustment of other medications was foreseen or the patient did not agree to taking BP preparations; (d) a second PSG was performed 2 months after starting BP treatment (still under treatment). The two PSGs were scored following the appropriate AASM Manual,^9^ allowing for a comparison of the outcomes before and after BP treatment. Figures 1 and 2 show representative examples of these pre‐/postcomparisons. Patients filled out several questionnaires about sleep quality and intensity of RLS symptoms twice: the first time during the first consultation or at the time point of the first PSG, and the second time after 2 months of BP treatment. In this way, the following indexes could be compared: Pittsburgh Sleep Quality Index, PSQI^10^, ^11^; Epworth Sleepiness Scale, ESS^12^; PLMS scale from the Douglass score^13^; and International RLS Severity Scale, IRLS.^14^ The main results of PSG and of the questionnaires are depicted in Tables 1 and 2, respectively. As so often in daily clinical practice, some of the patients had additional morbidities and several were taking medications; see Table 3 for medication overviews. One patient (patient 5) had a lactose intolerance. Although the amount of lactose in the 50% chewable tablets is low in comparison with the 10‐12 g lactose that can be tolerated by the majority of patients with this intolerance,^15^ she did not want to take the tablets. Since she insisted in trying a BP preparation, we suggested she take a BP 33% tincture (33% of fresh plant with 45% ethanol w/w; final ethanol content 36% v/v; DEV 0.04:1, ie 40 mg dried BP matter in 1 g mother tincture; Weleda AG, Arlesheim, Switzerland; used in a previous study^16^).

**Table 1:** Main polysomnography results before starting treatment with *Bryophyllum pinnatum* preparations and approximately 2 mo thereafter for each patient

	Total sleep time (min)	Sleep efficiency (%)	Sleep latency (min)	REM sleep (%)	REM sleep latency (min)	Sleep stages N1 and N2 (%)	Sleep stages N3 (%)	Arousal index (n/h)	Extremities movements (n/h)	PLMS (n/h)
Patient 1										
Before	307	64	1	5	254	76	19	22	12	6
After	431	90	1	6	373	77	17	20	6	1
Patient 2										
Before	312	87	2	10	322	83	7	8	83	76
After	363	76	2	9	348	72	20	13	52	41
Patient 3										
Before	416	86	1	17	155	72	11	16	17	8
After	337	70	0	18	96	70	12	25	28	16
Patient 4										
Before	315	67	9	10	82	63	26	78	99	88
After	332	88	20	16	100	72	12	21	78	73
Patient 5										
Before	442	97	29	21	74	60	18	31	34	30
After	457	96	13	23	113	64	13	27	25	20

No sleep‐associated breathing symptoms occurred (not shown).

PLMS, periodic limb movements in sleep; REM, rapid eye movement.

**Table 2:** Subjective outcomes at anamnesis and approximately two months after start of treatment with a *Bryophyllum pinnatum* preparation per patient

	PSQI^a^	ESS^b^	Douglass score—PLMS scale^c^	IRLS^d^
Patient 1				
Before	12	7	28	25
After	5	6	31	20
Patient 2				
Before	9	3	25	20
After	10	0	28	9
Patient 3				
Before	13	10	33	18
After	12	11	31	17
Patient 4				
Before	4	7	12	20
After	2	4	15	2
Patient 5				
Before	14	7	27	20
After	5	5	18	10

a PSQI (Pittsburgh Sleep Quality Index, 0‐15 scale, values higher than 5 indicate sleep disorders^10^, ^11^).

b ESS (Epworth Sleepiness Scale, 0‐24 scale, healthy persons often show values close to 6; values higher than 16 are indicative of a high level of daytime sleepiness^12^).

c Douglass score—PLMS.^13^ In the case of patient 4, one answer needed to calculate the Douglass score—PLMS before starting treatment was missing and was replaced by the average value of the remaining score items. Since no patient had Douglass scores indicative of sleep apnea, narcolepsy, or psychiatric sleep disorder, the corresponding data are not shown.

d IRLS (International RLS Severity Scale; 0‐40 scale; 0, none; 1‐10, mild; 11‐20, moderate; 21‐30, severe; 31‐40, very severe^14^).

**Table 3:** Medications taken by each patient at the start of treatment with a *Bryophyllum pinnatum* preparation

Patient	Medication (name, dose)
1	Acetylsalicylic acid, occasionally
	Antacid, occasionally
	L‐DOPA, 62.5 mg in acute phases of RLS only
	Magnesium, occasionally
	Rotigotine patch, 2 mg
	Tramadol, 10 drops in acute phases of RLS only
2	Amlodipine, 5 mg/d
	Bisoprolol, 2.5 mg/d
	Candesartan, 8 mg/d
	Cymarin, 30 drops/d
	Rivaroxaban, 20 mg/d
3	Doxylamine/valerian, 20 drops once per week or per 2 wk
4	‐
5	Citalopram, 20 mg/d
	Natalizumab, 300 mg iv every 4 wk
	Riboflavin, 200 mg/d

### 2.1 Patient 1

A 59‐year‐old woman presented with severe long‐term RLS after treatment with pramipexole and pregabalin, which had to be stopped due to side effects. At presentation, she was treated with rotigotine, a dopamine agonist in the form of a patch, with the additional possibility of taking L‐DOPA in acute phases of the disease and magnesium if needed. Baseline PSG showed low sleep efficiency (67%) with many arousals (22/h) and many short phases of wakefulness in which she frequently moved because of restlessness. During sleep, there were only few periodic leg movements (PLMS score 6/h) and light obstructive sleep apnea (apnea‐hypopnea index 7.5/h, oral desaturation index 8/h). She started add‐on treatment with BP 50% tablets at four tablets per day (0‐0‐2‐2) without changing other medication. As shown in Figure 1, PSG performed approximately 2.5 months thereafter and still under treatment revealed clear improvements in sleep architecture; sleep efficiency markedly improved (from 67% to 90%), PLMS index was very low (1/h). In her subjective feedback about this recorded night, the patient assessed her sleep as very good. In general, the patient reported a marked improvement of RLS symptoms and of the ability to sleep, but with severe phases of restlessness and sleepless nights still experienced from time to time.

### 2.2 Patient 2

A 65‐year‐old woman presented with severe insomnia and panic attacks, treated with trimipramine, a tricyclic antidepressant. Additionally, she was suffering from prediabetes and cardiovascular disease. Baseline PSG revealed disturbed sleep architecture with a high prevalence of periodic leg movements during sleep (PLMS index 76/h). An exact sleep history revealed that she suffered from restless legs in the evening and at night. The symptoms had started in late childhood and had slowly worsened over time. At the time of baseline PSG, RLS symptoms were quite bothering (IRLS 28/40). After the baseline PSG, the patient stopped trimipramine treatment spontaneously, which resulted in a reduction of RLS symptoms (IRLS decreased from 28/40 to 20/40). Then she started treatment with BP (50% chewable tablets, 0‐0‐3‐3). After 2 months of BP treatment, the patient described a significant improvement in sleep, less suffering from restless legs during the early part of the night, less nocturnal awakening, and greater restorative value in the morning. The patient seemed to benefit markedly from treatment with BP. The RLS improvement perceived by the patient was apparent in the change of her IRLS score (from 20/40 before treatment down to 9/40) and in the reduction of the PLMS index as calculated from the nocturnal PSG (from 76/h down to 41/h).

### 2.3 Patient 3

A 53‐year‐old woman presented with insomnia, likely related to psychophysiological factors for which she had been regularly taking the benzodiazepine medication lorazepam at a low dose. She had had sleep problems for 10 years and now complained of insomnia symptoms and was suffering from fatigue. She also suffered from RLS (IRLS 18/40), which had worsened under treatment with mirtazapine and improved with pramipexole in the past, but was currently not being treated. Once a week, she would stumble and suddenly fall down, events most probably caused by side effects of lorazepam. In her opinion, RLS complaints were not the main cause of her sleep disorder. During the first consultation, the patient was told to stop taking lorazepam, which she did. Four weeks later, the baseline PSG was performed, revealing markedly fragmented sleep architecture. Chewable tablets with 50% BP (six tablets per day; 0‐0‐3‐3) were recommended to the patient. After well‐tolerated treatment with BP for 2 months, no major changes neither in the objective PSG‐outcomes (see Table 1), nor in perceived insomnia, or restless leg discomfort were observed. The patient reported, however, that the course of symptoms had varied markedly: During BP treatment, the falling episodes became less frequent and she was able to continue without taking any benzodiazepine medication, which she viewed positively.

### 2.4 Patient 4

A 59‐year‐old man presented with severe sleep problems, low sleep quality, and daytime sleepiness. He did not have any medical history and was on no medication. PSG showed severely fragmented sleep architecture due to frequent periodic leg movements (PLMS score 88/h). A detailed sleep history revealed that he suffered from typical RLS (IRLS 20/40). He complained about waking up frequently during the night and having difficulties falling asleep again because of a disturbing restlessness in his legs. He was not taking any medication. Treatment with BP (50% tablets at four tablets per day; 0‐0‐2‐2 tablets) was initiated. Approximately two months later, the patient reported significant improvements in night sleep, no further RLS symptoms (IRLS 2/40) and better restedness in the morning. Comparison of the PSG outputs at before and after BP treatment revealed that sleep efficiency had improved (from 67% up to 88%), arousals and waking phases during the night had decreased (arousal index 21/h compared to 78/h before treatment), and the PLMS index slightly decreased (from 88/h to 73/h).

### 2.5 Patient 5

A 41‐year‐old woman presented with chronic insomnia that had lasted for years and had become worse in the last 1.5 years. In addition, she had had multiple sclerosis for approximately 9 years. About one year before, she was found to have RLS and her husband reported nocturnal leg movements, which could be PLMS. At presentation, the patient was being treated with the selective serotonin re‐uptake inhibitor (SSRI) citalopram for previous depression. According to the patient, RLS symptoms appeared about five times per week, mostly before falling asleep, sometimes also during awakening (IRLS 20/40). Her subjective sleep quality was low, which translated into a pathologically elevated PSQI; it was 12, whereas people without sleep disorders exhibit values up to 5. A PSG revealed disturbed sleep architecture, associated with a high PLMS index (30/h). After the PSG, BP treatment was suggested. Since the patient had a lactose intolerance, she did not want to take the 50% chewable tablets and was offered the possibility of taking a BP 33% tincture, which she accepted (at 0‐0‐20‐20 drops per day). After 2 months' treatment, the patient reported that she felt better during the day and that the RLS symptoms at night occurred only rarely (IRLS reduced from 20/40 to 10/40). This is in line with the lower PLMS index as measured during PSG (from 30/h to 20/h, see also Figure 2) and with the lower value of the PLMS scale from the Douglass questionnaire. Furthermore, her sleep quality had improved markedly (the PSQI went down to 4). When the patient forgot to take the BP medication (about one day every second week), she woke up between 2 and 3 o'clock and had difficulty in falling asleep again, even though she was in bed as usual between 11:00 pm and 6:00 am The fatigue typical for multiple sclerosis was still there, but the perceived tendency to fall asleep was less pronounced, she did not always need a daily nap and had more energy. After two months' treatment, the patient decided to continue to take BP 33% tincture. Fourteen months later, the patient was asked if she was still regularly taking BP 33% tincture; she was, and was still very positive about the effect.

## 3 DISCUSSION

*Bryophyllum pinnatum* (Lamarck) Oken [syn.: *Kalanchoe pinnata* (Lamarck) Persoon] is a plant of the family Crassulaceae. Though originally confined to Madagascar, BP now grows widely across tropical regions of Africa and America, India, China, and Australia. The plant is known under different common names, such as life plant, air plant, love plant, Cathedral bells, and Goethe plant. Leaf extracts from BP have been used in traditional medicine of the regions where it grows and are supposed to have, among other things, sedative and muscle relaxant properties.^17^, ^18^ In Europe, BP started to be used at the beginning of the 20th century in anthroposophic medicine, a form of holistic medicine with an integrative approach.^19^ The indications for which BP is often used within this type of medicine and more recently also in conventional settings include preterm labor, overactive bladder, and sleep disorders.^5^, ^6^ Several retrospective^5^, ^20^ and a few prospective studies on the treatment of preterm contractions^16^, ^21^ and of overactive bladder^22^ with BP preparations have been performed and suggest good effectiveness and very good safety/tolerability. The data are in line with the relaxant effects of BP preparations that have been observed in in vitro models, admittedly on smooth muscle, myometrium,^23^, ^24^ or detrusor.^25^, ^26^ Prospective observational studies have also revealed positive effects on sleep quality, both during pregnancy^7^ and among cancer patients.^8^ These clinical data are corroborated by in vivo experiments showing that different fractions of the leaf extract of BP can prolong the pentobarbitone‐induced sleeping time, indicating a CNS depressant action.^27^ This neurosedative effect could later be confirmed in a second study, which also revealed muscle relaxant effects.^18^ Some of the bufadienolides present in *Bryophyllum* species are thought to be responsible for the sedative effects.^28^, ^29^

The physician Willis first described a patient with RLS symptoms in 1685, while Ekbom used the term restless legs syndrome for the first time in 1945; therefore, RLS is also known as Willis‐Ekbom disease. The highly complex and even surprising pathophysiology of RLS (ICD‐10‐CM code: G25.81) has still not been fully elucidated. Genetic factors as well as elements of iron and dopamine neurotransmitter pathophysiology play major roles.^30^ It is assumed that regional brain iron deficiency activates hypoxic pathways, which in turn leads to increased dopaminergic activity and eventually to postsynaptic down‐regulation either at the receptor and/or at intracellular level. This latter postsynaptic deficit, together with the marked circadian dopamine activity pattern, triggers the typical RLS symptoms predominantly in the evening or at night. It is therefore understandable that a disturbed sleep profile and response to dopaminergic agents support the diagnosis of RLS. Also, PMLS and a positive familial history are supportive of this diagnosis. Prevalence rates lie between 3% and 12%.^2^, ^31^ Women are more affected than men are, especially during pregnancy, when prevalence is 2‐3 times greater than in the general population.^31^, ^32^ While first‐line agents such as dopamine agonists and α2δ calcium channel ligands relieve RLS, pronounced side effects can prevent their further (long‐term) use. Dopaminergic augmentation requires particular attention, as may be surmised from the pathophysiology described above and from the complex long‐term therapy algorithm that was recommended by a combined task force of various RLS‐related organizations.^3^ Therefore, alternative therapies are required. Several RLS features hamper treatment as well as the performance of clinical trials: (a) subjective assessment (how patients perceive the severity of their symptoms) may differ from the objective outcomes, for example PSG, (b) discontinuity of symptoms (there are sporadic daily changes in symptom intensity and many patients report singular phases of more or less severe symptoms), and (c) possible placebo effects. A meta‐analysis revealed an average placebo response rate of 40% in RLS trials. In this work, the highest placebo effect size was found for IRLS. However, for PLMS, the placebo effect was small, and for sleep efficiency, it was nearly absent.^33^

Our observations, obtained while experimentally treating five RLS patients with BP preparations, suggest that four patients have benefited from the treatment, albeit in different ways. The present consecutive case series was initiated because an improvement in sleep quality was to be expected on the basis of previous prospective observational studies.^7^, ^8^ Indeed, several improvements in sleep‐related outcomes were observed in this series. The pre‐/postcomparison of the PSGs reveals that the patients with low sleep efficiency (patient 1, female, and patient 4, male), moderate sleep latency (patient 5, female), and high arousal index (patient 4, male) at treatment begin could show improvement for these sleep parameters. The disease course of patient 4, who presented with severe sleep problems and restless legs symptoms, deserves to be highlighted. He was the only male patient in this prospective case series, was taking no additional medications, and showed marked objective improvement under BP. This included improvement of sleep efficiency (from 67% up to 88%) and fewer arousals (from 78/h to 21/h). Arousals reduction, which can be interpreted as a positive effect on sleep stability, was shown to occur in the course of RLS treatment with the benzodiazepine clonazepam.^34^ This strengthens earlier suggestions of the GABAergic effects of (some) BP components,^28^, ^29^ but future experimental research and randomized clinical trials would be required to confirm them. Interestingly, the patient who did not show improvements either in RLS symptoms or in sleep quality (number 3, female) was able to abstain from benzodiazepines. This favorably affected her health status, but rather due to benefiting from sedation without adverse events. Surprisingly, but in line with the previously described muscle relaxant effects of BP preparations, lowering the frequency of PLMS was observed in four out of five patients, most interestingly, also in the two patients with the highest PLMS indexes (patient 2, female, and patient 4, male). In general, analysis of the questionnaires filled out by the patients (mainly items used to calculate PSQI and IRLS) and patients' feedback supported those pre‐/postcomparisons of the PSGs. Taken together, the results of the present case series show that BP seems, as expected, to trigger sedative effects (several improvements in sleep‐related outcomes, fewer arousals), but also to cause some relaxing effects (fewer RLS symptoms, fewer periodic leg movements in sleep). Four out of the five patients showed relevant reductions of RLS symptoms, as was apparent in lower IRLS, an index that has been used as the gold‐standard outcome measure for clinical trials and was found to fulfill the criteria for “recommended” instruments to assess the severity of RLS symptoms (both in the practice setting and in clinical trials).^14^

In view of the present paucity of well‐tolerated and long‐term unproblematic treatments for RLS, our observations suggest that it might be worthwhile to treat RLS patients with (add‐on) BP preparations. These might be particularly indicated in the treatment of (frequent) RLS during pregnancy—especially if iron substitution is not needed or not enough—since the standard RLS medications are contra‐indicated in pregnancy^35^ and BP preparations are already recommended^36^ and in use for other indications.^21^ In a recent prospective observational study performed to assess BP prescriptions in the field of gynecology and obstetrics in Switzerland, one case showing a very good effect of BP medication on RLS during pregnancy was described.^21^ Moreover, several clinical trials revealed good tolerability during pregnancy.^7^, ^16^, ^20^

The main limitations of this case series are the heterogeneity of the patients, which potentially introduces a number of confounding factors, and the low number of PSGs performed for each patient (one before treatment and one thereafter). Given the variability of several RLS parameters from night to night, for example three PSG recordings at each occasion would have delivered more trustworthy results. Moreover, and in view of the patient collective, we had to depart from our original goal of including only patients without co‐medications in this case series, that is including patients who were not planning to change their medications. The co‐medications of four patients (only patient 4 was drug‐naïve) can be seen as an additional limitation. In the case of patient 2, the baseline PSG was performed still under trimipramine, a tricyclic antidepressant, which is presumed to worsen RLS 37; the BP contribution to the improvements of the PSG‐outcomes might therefore be overestimated.

The present case series shows that treatment of RLS with BP preparations deserves further clinical investigations and at the same time reveals several aspects to be considered in planning prospective efficacy trials with high patient numbers. The reduction of IRLS in four out of five patients suggests that this outcome is a valuable tool in assessing BP effects. Also, PSQI could be the appropriate tool for assessing changes in sleep quality in patients with RLS: In two patients, a clear‐cut PSQI reduction was observed that was even superior to that seen in the earlier prospective study on cancer patients (from 12 to 9 during 3‐week treatment^8^). Objective PLMS assessment in a prospective trial with ankle‐actigraphy would be an ideal, but straightforward, complement to IRLS and PSQI indexes. Since some effects might not be assessed with these two indexes, a (health‐related) quality of life questionnaire might be a useful complement. Finally, a trial on BP as an add‐on medication for RLS (in the absence of pregnancy) as well as one on BP as a single medication for pregnancy‐related RLS appears promising. In both cases, a solution for patients reluctant to ingest lactose might be worthwhile.

## 4 CONCLUSION

The disease courses of four out of our five patients suggest that RLS treatment with BP preparations (mainly chewable tablets, or the lactose‐free alternative, 33% mother tincture) can improve RLS and sleep quality in patients with RLS. Spasmolytic properties and the sedative effect of BP preparations seem to play a role. Given its excellent profile in terms of side effects, BP could also be used in pregnant women with RLS, where nonmedication treatment is considered primary if no iron treatment is needed or iron treatment alone is not successful. Prospective observational studies with high patient numbers and longer treatment duration are urgently needed.

## CONFLICT OF INTEREST

MS: is an employee of Weleda AG, the company that produces the preparations of *Bryophyllum pinnatum* used in this case series. Weleda AG: supported part of the costs. APSW: has received research funding from Weleda AG over the last five years.

## AUTHOR CONTRIBUTION

DF, UM, and APSW: conceived the present work. DF and BGS: treated one patient each; SM treated three patients. APSW: coordinated the series and wrote the first version of the manuscript. MS and UM: went through the manuscript critically. All authors reviewed the manuscript, then read and approved the submitted version.

## ETHICAL APPROVAL

The need for an ethics approval for a case series of no more than five patients was waived by the ethics committee of Kanton St. Gallen.

Consent to participate: The consent for treatment and publication was obtained from all five patients.
